# Nfatc2 and Tob1 Have Non-Overlapping Function in T Cell Negative Regulation and Tumorigenesis

**DOI:** 10.1371/journal.pone.0100629

**Published:** 2014-06-19

**Authors:** Sarah L. May, Qing Zhou, Mitzi Lewellen, Cristan M. Carter, David Coffey, Steven L. Highfill, Christoph M. Bucher, Ilze Matise, Herbert C. Morse, M. Gerard O’Sullivan, Melissa Schutten, Charles Johnson, Donald Bellgrau, Bruce R. Blazar, Jaime F. Modiano

**Affiliations:** 1 Masonic Cancer Center, University of Minnesota, Minneapolis, Minnesota, United States of America; 2 Department of Veterinary Clinical Sciences, College of Veterinary Medicine, University of Minnesota, St Paul, Minnesota, United States of America; 3 Department of Pediatrics, School of Medicine, University of Minnesota, Minneapolis, Minnesota, United States of America; 4 Integrated Department of Immunology, School of Medicine, University of Colorado, Denver, Colorado, United States of America; 5 University of Colorado Cancer Center, Aurora, Colorado, United States of America; 6 Department of Veterinary Population Medicine, College of Veterinary Medicine, University of Minnesota, St Paul, Minnesota, United States of America; 7 National Institute of Allergy and Infectious Diseases, NIH, Rockville, Maryland, United States of America; New York University, United States of America

## Abstract

Nfatc2 and Tob1 are intrinsic negative regulators of T cell activation. *Nfatc2*-deficient and *Tob1*-deficient T cells show reduced thresholds of activation; however, whether these factors have independent or overlapping roles in negative regulation of T cell responses has not been previously examined. Here, we show that *Nfatc2* knockout (KO) but not *Tob1* KO mice have age-associated accumulation of persistently activated T cells *in vivo* and expansion of the CD44^+^ memory cell compartment and age-associated lymphocytic infiltrates in visceral organs, without significant changes in numbers of CD4^+^CD25^+^Foxp3^+^ regulatory T cells (Treg). *In vitro*, CD4^+^CD25^−^ “conventional” T cells (Tconvs) from both KO strains showed greater proliferation than wild type (WT) Tconvs. However, while Tregs from *Nfatc2* KO mice retained normal suppressive function, Tregs from *Tob1* KOs had enhanced suppressive activity. *Nfatc2* KO Tconvs expanded somewhat more rapidly than WT Tconvs under conditions of homeostatic proliferation, but their accelerated growth capacity was negated, at least acutely, in a lymphoreplete environment. Finally, *Nfatc2* KO mice developed a previously uncharacterized increase in B-cell malignancies, which was not accelerated by the absence of *Tob1*. The data thus support the prevailing hypothesis that Nfatc2 and Tob1 are non-redundant regulators of lymphocyte homeostasis.

## Introduction

Since the unexpected phenotype of nuclear factor of activated T-cells-cytoplasmic component-2 (Nfatc2)-deficient mice was originally reported with excess peripheral lymphocytes, the mechanisms responsible for the expansion of these cells have become better understood [1]–[5]. Nfatc2 is a transcription factor with pleotropic functions. In combination with activation protein-1 (AP-1) transcription factors, Nfatc2 promotes expression of cytokines and other genes associated with lymphocyte activation. However, when Nfatc2 is activated by signals that fail to induce productive AP-1 assembly, it modulates transcription of a constellation of genes that make cells unresponsive to growth signals and promote cell cycle arrest [5].

Recent efforts to identify Nfatc2 partners that mediate these responses indicate that Nfatc2 can combine with Foxp3, and that these interactions, which also are modulated by other transcription factors such as Smad3 and Runx family members, are essential for complete induction and function of Foxp3 itself in CD4^+^ Tregs [6]–[8]. The available data support an important cell-intrinsic role for NFAT family transcription factors in intrinsic negative T cell regulation. For example, Nfatc2 KO mice are resistant to suppression of the G0 to G1 transition by nicotine [9], Nfatc2/Nfatc3-doubly deficient CD4^+^ Tconv cells are refractory to the effects of WT Treg cells in vitro [10], Nfatc2-deficient T cells resist tumor-induced anergy and promote tumor rejection [11], and mice with T cells harboring a hyperactivatable form of Nfatc2 show attenuated autoimmune responses to myelin basic protein [12]. However, the observation that oxazolone-induced ulcerative colitis and progression to colon cancer are attenuated in *Nfatc2* KO mice due to ineffective production of IL-6 [13], [14] suggests that the function of Nfatc2 is not uniquely to repress cell cycle or lymphocyte activation, but rather, it can act as a more general modulator of inflammation and even as an oncogene in non-lymphoid cells.

Regarding the role of Nfat family members in Treg development and function, it is likely that these also will be dependent on the context of both genetic and microenvironment factors. Specifically, *Nfatc2* KO mice in a BALB/c background produced greater numbers of inducible Treg cells (iTregs) than their WT counterparts in response to allergen-induced experimental asthma [15]. On the other hand, in a C57BL/6 background, the total mass of NFAT proteins (including Nfatc1, Nfatc2, and Nfatc3) was more important for development of iTreg cells than the contribution of any one family member [16]. However, Nfat activity seemed to be dispensable for Treg function in a model of autoimmune colitis [16].

Similar to *Nfatc2*, the transducer of ErbB2–1 (Tob1) also is a cell-intrinsic negative regulator of proliferation [17]. In T cells, Tob1 enforces quiescence by supporting expression of the CDK inhibitor, p27/Kip-1, and silencing the IL-2 promoter, as well as by modulating the activity of SMAD transcription factors [18]. Endogenous Tob1 is stabilized by partial antigen stimulation with altered peptide ligands, resulting in anergy [19]. The only phenotype associated with Tob1 deficiency is increased bone mass [20]; this genotype does not produce gross immune defects, but T cells from *Tob1* KO mice have a reduced threshold of activation *in vitro* and *in vivo* [4], [21], and in an experimental autoimmune encephalomyelitis model, *Tob1* KO cells showed greater CNS inflammation with increased infiltrating CD4^+^ and CD8^+^ T cells, increased myelin-reactive Th1 and Th17 cells, and reduced numbers of Tregs [22]. Thus, Tob1 appears to augment some types of Tconv effector function, while reducing Treg numbers.

The possibility of modulating Nfatc2 and Tob1 molecules to achieve therapeutic benefits, for example, as part of strategies to enhance T cell function by inhibiting Treg activity or by re-establishing adaptive T cell immunity in lymphodepleted patients remains unclear, and mouse models can provide important gating and feasibility data for such strategies. It is similarly not know if Nfatc2 and Tob1 exert redundant effects of Treg numbers and function in any species. Here, we sought to further clarify if there was redundancy in the function of Nfatc2 and Tob1 as cell-intrinsic negative regulatory factors and as extrinsic mediators of Treg activity.

## Materials and Methods

### Animals

Congenic *Nfatc2* KO, CD45.2 mice on the C57BL/6 (B6) H-2^b^ background were derived from B6×129/SvJ KOs (a kind gift of Dr. Anjana Rao, Harvard University and La Jolla Institute for Allergy and Immunology) back-crossed for 8 generations to WT B6 mice (Jackson Laboratory, Bar Harbor, ME) using a speed congenic approach [21]. Subsequently, the B6-*Nfatc2* KO mice were bred as homozygous knockouts. *Tob1* KO mice (derived from B6 ES cells in the H-2^b^ background, [20]) were kindly provided by Dr. Tadashi Yamamoto (The Institute of Medical Science, The University of Tokyo, Tokyo, Japan). *Tob1* KO mice have been deposited for distribution at the Jackson Laboratories with permission from RIKEN BioResource Center (Ibaraki, Japan). B6-*Nfatc2* KO mice were used for experiments after the 8^th^ generation when there were neither detectable haplotype differences nor evidence of one-way or two-way mixed lymphocyte reactivity between wild type B6 and *Nfatc2* KO spleen cells. Genotyping was confirmed using the services from Transnetyx (Cordova, TN) to maintain both strains. Pups from homozygous KO X KO *Tob1* matings were viable, but the females were extremely prone to dystocia and almost always failed to produce sufficient milk for the pups (see below). Mating strategies to produce *Tob1* KO mice included breeding heterozygous males to homozygous females, which resulted in smaller pups, and using foster dams to raise the litters as needed. Heterozygous matings also were used to generate hemizygous (*Nfatc2^+/−^*; *Tob1^+/−^*) and WT littermate controls. *Nfatc2/Tob1* double KO (DKO) mice were generated by breeding *Nfactc2* KO females to *Tob1* heterozygous males.

### Breeding Strategy and Phenotype of Nfatc2 X Tob1 DKO Mice

*Nfatc2* homozygous male mice were bred to *Tob1* heterozygous female mice to generate double heterozygous F1 pups. Eight F2 matings resulted in 40 pups (39 live and one dead) with an approximately Mendelian distribution, including 2 *Nfatc2 x Tob1* DKO pups (1 live and 1 dead). To increase the frequency of DKOs, we set-up an additional 10 matings between *Nfatc2* ^−/−/^ *Tob1^+/−^* pairs, which produced 47 pups. Only 5 pups from these matings were *Nfatc2^−/−/^Tob1^−/−^* (DKOs), although they were all viable; 17 were *Nfatc2^−/−/^Tob1^+/+^* and 25 were *Nfatc2^−/−/^Tob1^+/−^*. The small litter sizes and the non-Mendelian distribution, especially in the *Nfatc2^−/−/^Tob1^+/−^* matings suggests that the DKO phenotype might have detrimental effects during development.

The *Nfatc2^−/−/^Tob1^+/−^* dams generally failed to produce milk, so pups were fostered on CD-1 dams. Histologically, adult female Tob^+/−^ and *Nfatc2^−/−/^Tob1^+/−^* mice showed comparable mammary development as wild type B6 mice, indicating the inability to nurse their pups was not due to an anatomic defect in mammary development. *Nfatc2^−/−/^Tob1^+/−^* mice have been deposited for distribution at the RIKEN BioResource Center (Ibaraki, Japan).

For all strains, “young” mice were <12 weeks of age and “old” mice were >15 months of age. All animals were maintained in specific pathogen-free conditions.

### Ethics Statement

This study was carried out in strict accordance with recommendations in the Guide for the Care and Use of Laboratory Animals of the National Institutes of Health. Protocols were approved by the University of Minnesota Institutional Animal Care and Use Committee (protocol #s 1309-30918A, 1010A90715, 0901A56883, 0712A23201).

### Flow Cytometry and Antibodies

Cells were stained using routine methods [21], and analysis was done on the FACSCalibur flow cytometry platform (BD Biosciences, San Jose, CA). Intracellular staining was done using perm/fix solution from eBioscience (San Diego, CA) following the manufacturer’s recommendations. Antibodies against CD3 (145-2C11), CD4 (GK1.5), CD8 (53-6.7), CD44 (IM7), CD69 (H1.2F3), B220 (RA3-6B2), CD19 (MB19-1), CTLA-4 (UC10-4B9), CD11b (M1/70), CD11c (N418), NK1.1 (PK136), Foxp3 (FJK-16s), and CD25 (PC61.5 and eBio7D4) were obtained from eBioscience. Anti-CDK4 (C22) was obtained from Santa Cruz Biotechnology (Santa Cruz, CA). Data were analyzed by FlowJo software version 8.87 (Treestar, Ashland, OR).

### Pathological Examination

Microscopic assessment of tissue sections was done by HCM and board-certified veterinary pathologists (IM, MGO, MS, and CJ). Histologic diagnoses of hematopoietic neoplasms were made based on established criteria [23]. H&E and immunohistochemical staining was performed by the staff of the comparative pathology shared resource (CPSR) of the Masonic Cancer Center, University of Minnesota.

### Enrichment of Conventional and Regulatory T Cell Subsets

CD4^+^ T cells were isolated from single cell suspensions of spleens and lymph nodes from WT, *Nfatc2* KO and *Tob1* KO mice by negative immunomagnetic selection using the Miltenyi Biotec (Auburn, CA) microbead platform following the manufacturer’s instructions. Immunomagnetic depletion of non-CD4^+^ T cells was accomplished using a cocktail of antibodies against CD11c (N418), CD11b (M1/70), NK1.1 (PK136), CD8 (53-6.7), and B220 (RA3-6B2). Following depletion of non-CD4^+^ T cells, CD25^+^ cells were enriched by positive selection using anti-CD25-biotin (eBio7D4), and collecting the flow-through enriched for CD4^+^CD25^−^ cells, hereafter called “Tconvs”. Stimulator cells (antigen presenting cells or AgPCs) were enriched from spleen cells by depletion of T cells and NK cells using antibodies against CD3 and NK1.1. Preparations used for experiments consisted of >95% pure CD4^+^CD25^+^ or CD4^+^CD25^−^ cells as determined by flow cytometry. For enrichment of CD44^bright^ and CD44^dim^ subsets, CD4^+^CD25^−^ cells were purified as above, labeled with anti-CD44 antibody conjugated to allophycocyanin, and sorted on a FACSAria fluorescence activated cell sorter (BD Biosciences).

### Cell Labeling

Loading with 5-(and-6)-carboxyfluorescein diacetate, succinimidyl ester (CFSE, Invitrogen, Carlsbad, CA) was done by incubating cells in a 1 µM CFSE solution in PBS for 2 minutes at room temperature, stopped by addition of fetal bovine serum (FBS, Atlas Biologicals, Fort Collins, CO). PKH26 (Sigma-Aldrich St. Louis, MO) was diluted 500 times using the conditions recommended by the manufacturer, incubated with cells for 3 minutes at room temperature, and stopped by addition of media as above. Dye-loaded cells were washed thoroughly in PBS before use in experiments.

### T Cell Proliferation and Suppression Assays

Mixtures of 1×10^5^ CFSE-labeled Tconv CD4^+^CD25^−^ and 1×10^5^ stimulator accessory cells with or without anti-CD3 antibody (1 ng/ml) were incubated in triplicate wells of 96-well U-bottom plates. 5×10^4^ PKH26-labeled, CD4^+^CD25^+^ Tregs were added at the onset of experiments as indicated in the results. Cells were incubated in a humidified atmosphere containing 5% CO_2_ at 37°C in RPMI 1640 supplemented with 10% FBS for 4 days, after which proliferation was assessed by CFSE dilution. Cyclosporin A (CsA) (1 µg/ml) was added to Treg cells for 1 hr, after which the cells were washed thoroughly and mixed with Tconv cells for the CFSE assays. CsA treatment did not affect Treg cell viability based on trypan blue exclusion.

### Adoptive Transfers and In vivo Expansion

1×10^5^ age matched, differentially CFSE-labeled WT and/or *Nfatc2* KO CD4^+^CD25^−^CD45.2 cells were injected intravenously into lymphoreplete B6-CD45.1 or lymphopenic B6-SCID mice. After 15 days, lymph nodes and spleen cells were collected and analyzed for total CD4^+^ T cell expansion based on CD4^+^CD45.2^+^ cell numbers and CFSE dilution. Cells were enumerated using a CellDyn 3500 automated cell counter. Percentages were calculated based on flow cytometry.

### Statistics

Descriptive statistics (mean, median, standard error, standard deviation) were generated using Microsoft Excel and GraphPad Prism. Student t-test was used for pairwise comparisons. A p value<0.05 was used as the determinant for significant differences between samples.

## Results

### Nfatc2 KO, but not Tob1 KO Mice have an Increased Frequency of Persistently Activated Lymphocytes In vivo

**Figure 1:**
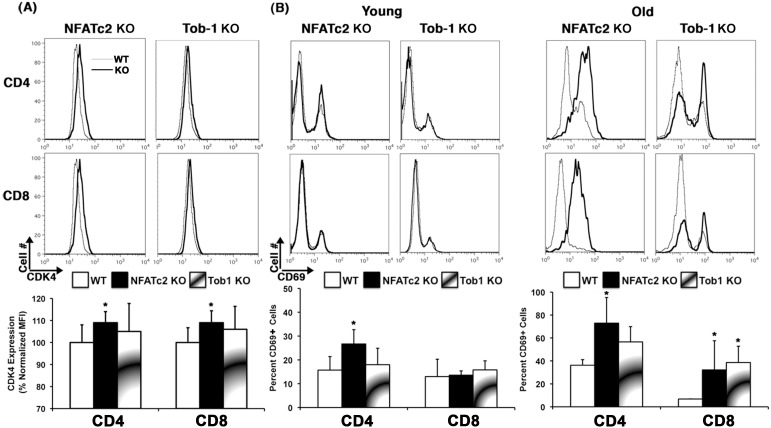
*Nfatc2* and *Tob1* KO mice have increased numbers of persistently activated cells *in vivo*. Spleen and lymph node cells were isolated from age-matched WT, *Nfatc2* KO, and *Tob1* KO mice, and cells from each genotype and for each organ were pooled for experiments. Expression of CDK4 was measured by intracellular staining and expression of CD69 was measured by conventional cell surface staining of freshly isolated cells. (A). One-dimensional histograms (top) showing CDK4 expression, gated on CD4 and CD8 cells from representative *Nfatc2* KO and *Tob1* KO mice overlaid on WT controls as indicated. Dark lines in the histograms represent KOs and grey lines represent WT mice. Bar graphs (bottom) represent means ± SD of the mean fluorescence intensity (MFI) for CDK4 expression in CD4 and CD8 cells. Data summarize 15, 12, and 5 experiments using triplicate samples of WT cells, *Nfatc2* KO cells, *Tob1* KO cells, respectively, each with pooled cells from 2 or 3 mice. MFIs among different experiments showed normal distribution. Asterisks denote values that are significantly different from WT (Student t-test p<0.05). (B) One-dimensional histograms (top) showing CD69 expression, gated on CD4 and CD8 cells from representative young (left) and old (right) *Nfatc2* KO and *Tob1* KO mice overlaid on WT controls as indicated. Dark lines in the histograms represent KOs and grey lines represent WT mice. Bar graphs (bottom) represent means ± SD percent CD69^+^ cells in the CD4 and CD8 compartments. Data for young and old mice summarize 10 and 4 experiments using triplicate samples of WT cells, 6 and 8 experiments using triplicate samples of *Nfatc2* KO cells, and 4 and 3 experiments using triplicate samples of *Tob1* KO cells, respectively. The percent of positive cells among different experiments showed normal distribution. Asterisks denote values that are significantly different from WT (Student t-test p<0.05).

While a hallmark of the *Nfatc2* KO genotype is expansion of T cells within peripheral lymphoid organs, a similar phenotype was not grossly apparent in *Tob1* KO mice [20]. Previously, we showed that the capacity of Tconvs from *Nfatc2* KO mice to exit cell cycle is impaired [24]. We surmised this would cause age-dependent accumulation of persistently activated memory T cells in secondary lymphoid organs of *Nfatc2* KO mice, but not *Tob1* KO mice. To evaluate this, we quantified activated T cells (CDK4^+^ and CD69^+^) in spleen and lymph nodes from WT, *Nfatc2* KO, and *Tob1* KO mice using flow cytometry. As shown previously [9], [24], CD4^+^ and CD8^+^ T cells from *Nfatc2* KO mice showed a modest, but significant elevation of CDK4 expression (Figure 1A, ∼10% increase in MFI), which was seen in unperturbed cells from 10/12 young mice tested when compared to 15 age-matched WT mice. In contrast, CDK4 expression in T cells from *Tob1* KO mice was variable. It was elevated in 2/7 mice, and equal to or below the levels of WT mice in 5/7 animals tested. Accumulations of CD69^+^ cells were observed as a subtle change in young *Nfatc2* KO mice, but became quite pronounced in old mice (Figure 1B). These cells reproducibly accounted for ∼70% of CD4^+^ T cells and almost 40% of CD8^+^ T cells in old *Nfatc2* KO mice, and for >50% of CD4^+^ T cells and almost 40% of CD8^+^ T cells in old *Tob1* KO mice, as compared to ∼30% and <5%, respectively, in WT mice.

### Nfatc2 KO, but not Tob1 KO Mice Show Age-related Accumulations of Memory T Cells

**Figure 2:**
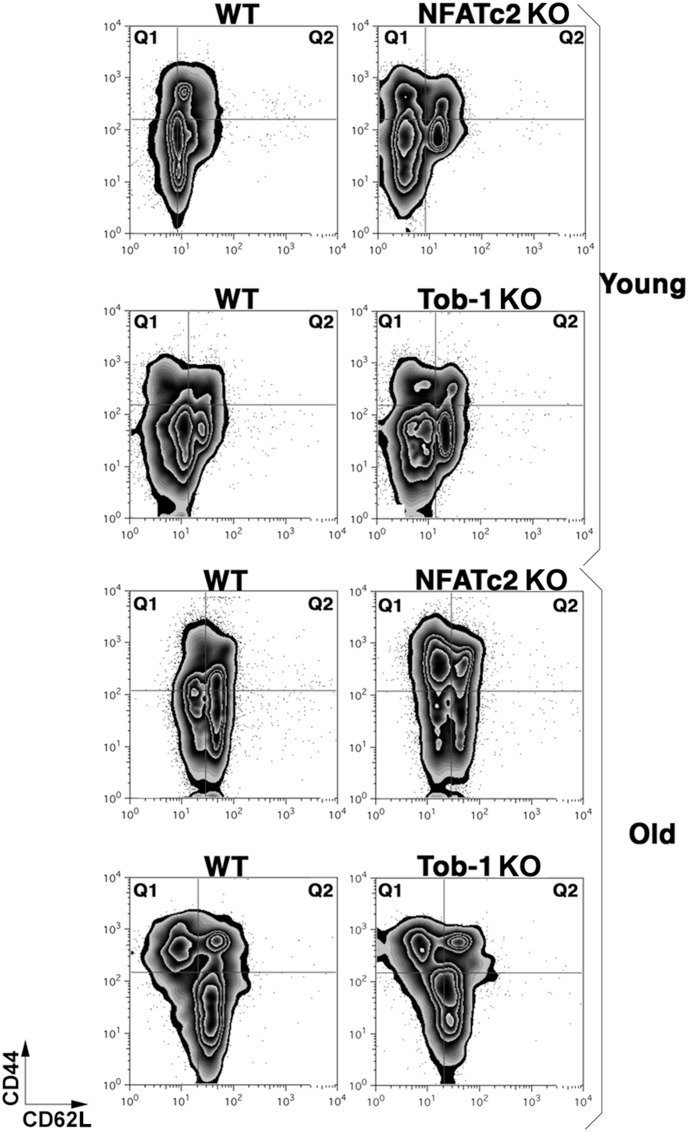
*Nfatc2* KO mice, but not *Tob1* KO mice show age-related accumulation of memory T cells. (A) Spleen and lymph node cells were isolated from age-matched WT, *Nfatc2* KO, and *Tob1* KO mice, and cells from each genotype and for each organ were pooled for experiments. Expression of CD44 and CD62L was measured by conventional cell surface staining in WT, *Nfatc2* KO, and *Tob1* KO T cells immediately after isolation from spleens or lymph nodes. Panels are representative two-dimensional contour plots showing CD44 and CD62L staining from young (top) and old (bottom) mice gated on CD3 T cells from representative mice as indicated. Similar data for young and old mice were obtained in 9 and 4 experiments using WT cells, 6 and 4 experiments using *Nfatc2* KO cells, and 4 and 4 experiments using *Tob1* KO cells, respectively. Means ± SD are provided in Table 1.

Our results indicated that *Nfatc2* deficiency led to a progressive increase in the frequency of cells with a persistently activated phenotype even at a young age. This change was protracted in *Tob1* deficient animals, which exhibited a reduced frequency of activated T cells in comparison to old, age-matched *NFATc2* KOs. One explanation for these differences would be that *Nfatc2* KO, but not *Tob1* KO T cells can readily transition to a memory phenotype and persist in peripheral lymphoid tissues. To test this directly, we examined expression of CD44 and CD62L in spleen and lymph node T cells from WT (8 young and 4 old), *Nfatc2* KO (7 young and 4 old), and *Tob1* KO (4 young and 3 old) mice. Figure 2 and Table 1 show that increased frequencies of CD44^bright^ memory T cells were not seen in *Nfatc2*-deficient young mice or in *Tob1*-deficient vs. WT control mice, but CD44^bright^CD62L^dim^ (Tconv memory) T cells were significantly increased in old *Nfatc2* KO, but not *Tob1* KO vs. WT control animals. A similar increase in the frequency of memory cells was seen in the B cell compartment of *Nfatc2* KO mice as compared to WT mice.

**Table 1:** Means (± SD) of the percent CD44^bright^CD62L^dim^ and CD44^bright^CD62L^bright^ for WT, Nfatc2 KO, and Tob1 KO T cells from young and old mice.

Age and Genotype	% CD44^bright^CD62L^dim^ (Mean ± SD)	% CD44^bright^CD62L^bright^ (Mean ± SD)
Young WT	9.04±3.9	11.8±6.3
Young Nfatc2 KO	13.1±4.1	10.6±5.0
Young Tob1 KO	8.70±0.9	8.49±4.8
Old WT	20.8±12.6	16.2±5.1
Old Nfatc2 KO	49.8±20.4	17.4±8.1
Old Tob1 KO	30.4±4.9	19.4±4.2

Data were derived from 9, 6, and 4 young WT, *Nfatc2* KO, and *Tob1* KO mice, respectively, and from 4, 4, and 4 old WT, *Nfatc*2 KO, and *Tob1* KO mice, respectively.

### T Cell Expansion in Nfatc2 and Tob1 Knockout Mice is not Due to Altered Treg Numbers

**Figure 3:**
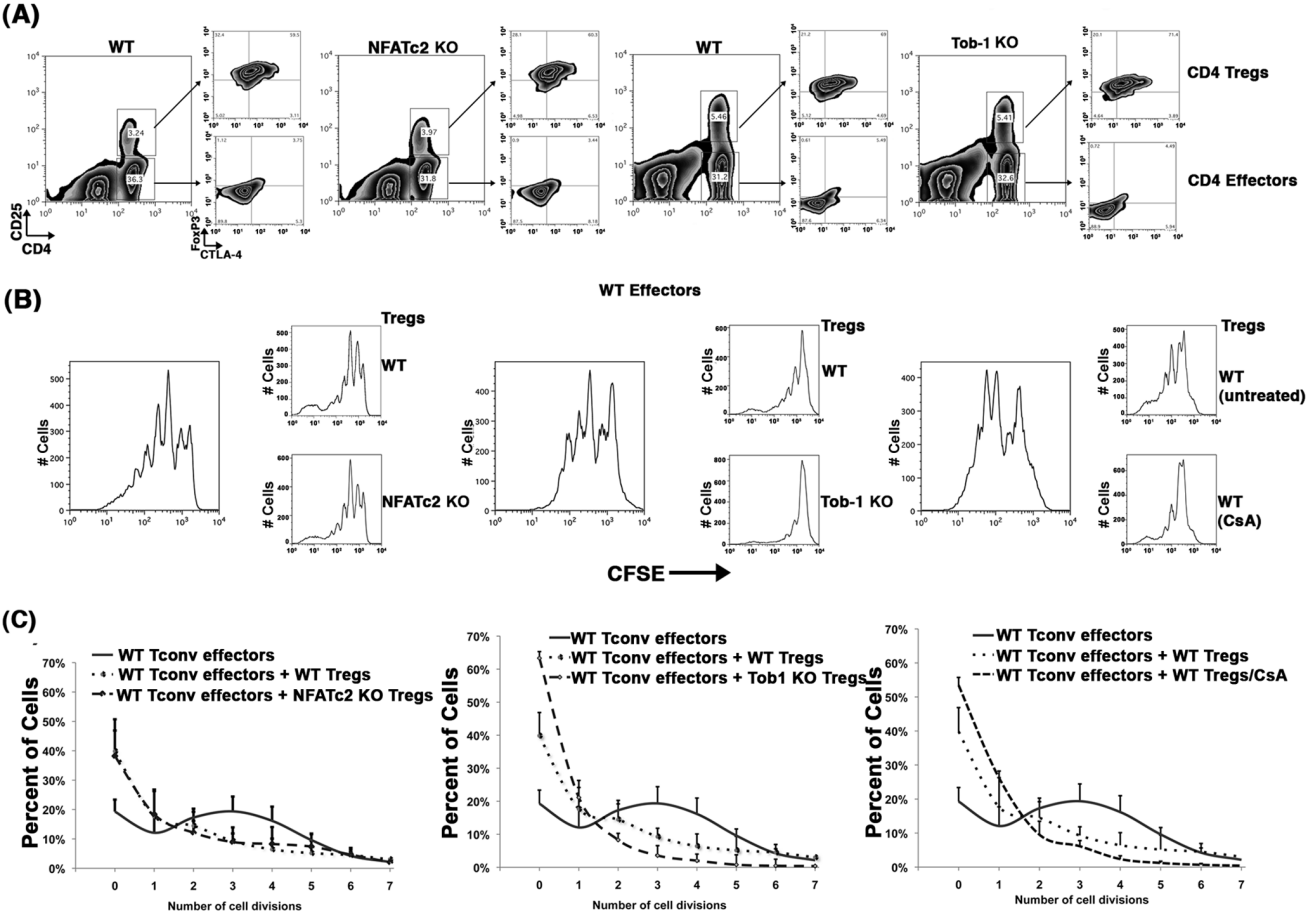
T cell expansion in *Nfatc2* and *Tob1* KO mice is not due to Treg dysfunction. CD4 T cells were isolated from single cell suspensions of spleens and lymph nodes from age-matched WT, *Nfatc2* KO, and *Tob1* KO mice by negative immunomagnetic selection. CD4^+^CD25^−^ (Tconv) cells and CD4^+^CD25^+^ (Tregs) were enriched by sorting. Tconv cells were labeled with CFSE and Treg cells were labeled with PKH26. Tconvs (100,000/well) were then mixed 1∶1 with syngeneic AgPCs and stimulated with anti-CD3 in the presence of absence of 50,000 Treg cells as indicated. Proliferation was measured by CFSE dilution in CD4 T cells using flow cytometry after of 96 hr of culture. (A) Expression of CD4, CD25, and CTLA-4 was assessed by conventional cell surface staining; expression of Foxp3 was examined by intracellular staining Panels are representative two-dimensional contour plots showing CD4 and CD25 staining from WT, *Nfatc2* KO, and *Tob1* KO mice gated on CD3 T cells. Boxed areas represent CD4^+^CD25^+^ (Treg) cells and CD4^+^CD25^−^ (Tconv) cells. Small panels to the right represent two-dimensional contour plots showing CTLA-4 and Foxp3 staining, respectively for Tregs on top and Tconvs below. (B) Representative one-dimensional histograms of CFSE dilution from stimulated WT cells. Small panels to the right represent one-dimensional histograms of CFSE dilution for the same WT cells, respectively with the addition of WT Tregs, *Nfatc2* KO Tregs, *Tob1* KO Tregs, or WT Tregs treated with CsA as indicated. (C) Means ± S.D. of the percent of cells that underwent 0–7 divisions over 96 hr for each genotype with and without Tregs. The data summarize 8 experiments using WT Tregs, 4 experiments using *Nfatc2* KO Tregs, 2 experiments using *Tob1* KO Tregs, and 2 experiments using WT Tregs treated with CsA, each with pooled cells from 2 or 3 mice. The number of WT Tconv cells that did not undergo cell division was significantly greater (p<0.01), and the number of cells undergoing 2 or more divisions was significantly reduced (p<0.0001) when *Tob1* KO Tregs were present in the culture than when WT Tregs or *Nfatc2* KO Tregs were present in the culture.

The role of *Tob1* in Treg cells has not yet been examined. To address this issue, we first compared the frequencies of CD4^+^CD25^+^Foxp3^+^ cells in peripheral lymphoid organs of WT, *Nfatc2* KO, and *Tob1* KO mice. Figure 3A shows that young *Nfatc2*-deficient and *Tob1*-deficient mice have the same proportions of peripheral Tregs (CD4^+^CD25^+^) as their WT littermate controls, and while there was erosion of Tregs with age in general, this process was not accelerated in *Nfatc2* KOs or in *Tob1* KOs (not shown). Tregs from *Nfatc2* KO and *Tob1* KO mice expressed similar levels of Foxp3 or CTLA-4 (Figure 3A, insets) as well as IL-6R (data not shown).

### Nfatc2 and Tob1 KO Tconvs are More Easily Activated In vitro

**Figure 4:**
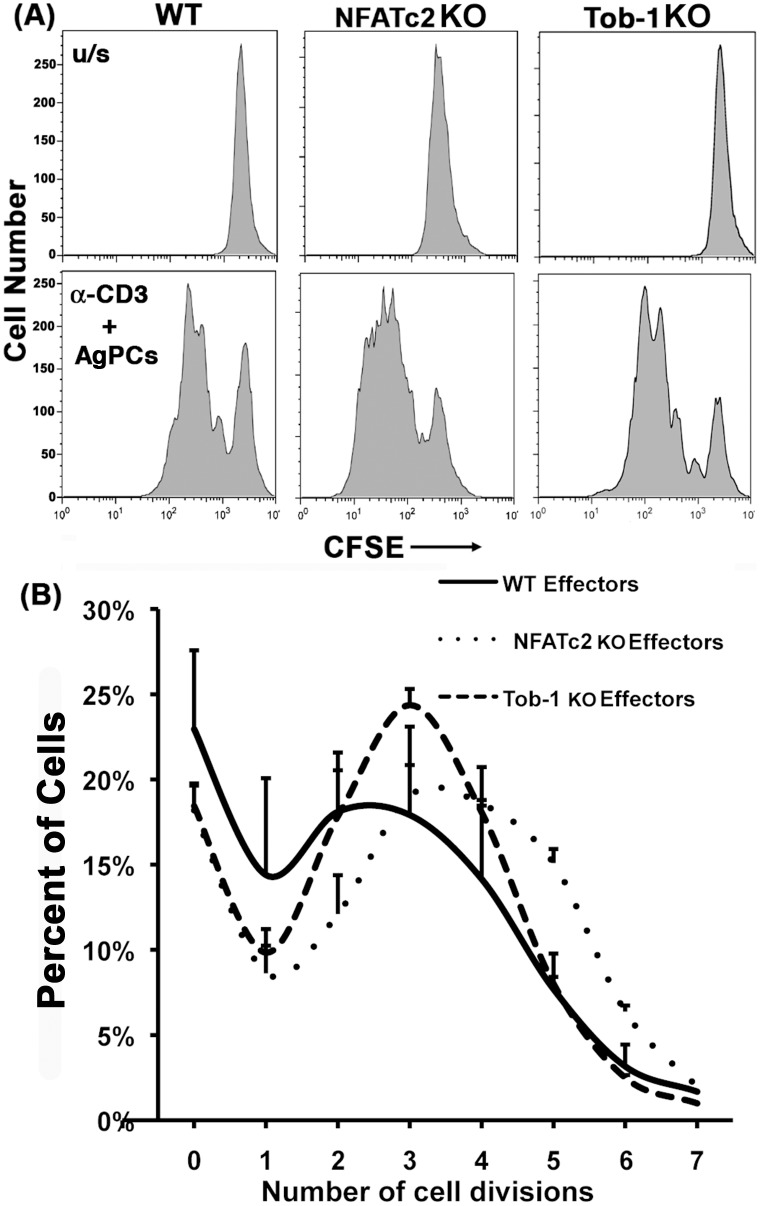
*Nfatc2* and *Tob1* KOs lead to hyper-proliferative T-cell responses *in vitro*. CD4 T cells were isolated from single cell suspensions of spleens and lymph nodes of age-matched WT, *Nfatc2* KO, and *Tob1* KO mice by negative immunomagnetic selection. CD4^+^CD25^−^ (Tconv) cells were then enriched by depletion of CD4^+^CD25^+^ (Treg) cells and labeled with CFSE. Tconv cells were mixed 1∶1 with syngeneic CD19^+^ spleen cells (AgPCs) and stimulated with anti-CD3 (1 ng/ml). Proliferation was measured by CFSE dilution in CD4 T cells using flow cytometry after of 96 hr of culture. (A) Representative one-dimensional histograms of CFSE dilution from unstimulated (top) or from stimulated (bottom) WT, *Nfatc2* KO and *Tob1* KO T cells from one experiment using pooled spleen cells from 3 mice each. (B) Means ± S.D. of the percent of cells that underwent 0–7 divisions over 96 hr for each genotype. The data summarize 8, 4, and 3 experiments using triplicate samples of WT cells, *Nfatc2* KO cells, and *Tob1* KO cells, respectively, each with pooled cells from 2 or 3 mice.

There was a qualitative difference between the *Nfatc2* KO and the *Tob1* KO T cell phenotypes. Figure 4A shows T cell proliferation assays with CFSE labeled Tconvs activated *in vitro*. There were similar percentages of non-dividing T cells after anti-CD3 stimulation of WT cells, *Nfatc2* KO cells, and *Tob1* KO cells. Figure 4B shows a comparison of the number of cell divisions achieved by WT, *Nfatc2* KO, and *Tob1* Tconvs after 96 hr in culture. Proliferation of *Tob1* KO cells peaked after 3 rounds of cell division, whereas *Nfatc2* KO cells showed robust cell division for 4–5 cycles. Approximately twice as many *Nfatc2* KO T cells underwent >4 rounds of division than T cells from WT or *Tob1* KO mice, consistent with the notion that *Nfatc2* deficiency has great impact on T cell cycle exit and/or accumulation of memory cells with shorter doubling times [24].

### Naïve T Cells from Nfatc2 Knockout Mice Exhibit a Hyper-proliferative Phenotype

**Figure 5:**
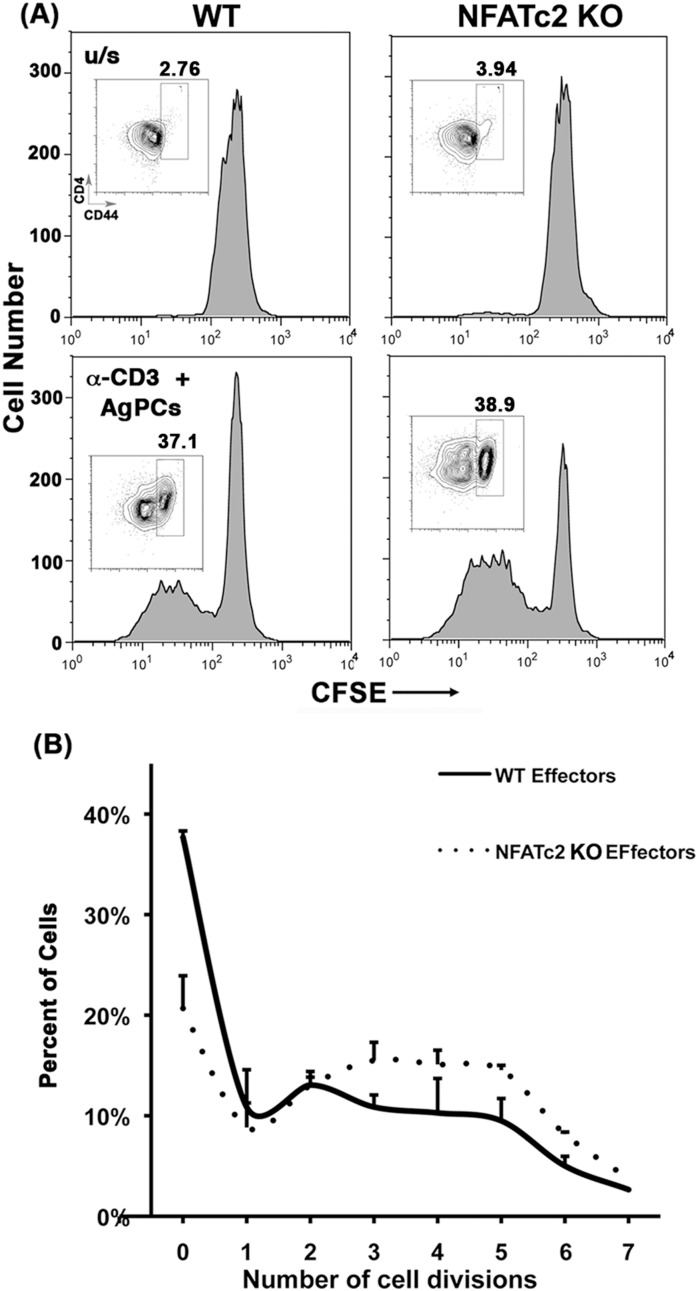
Naïve T cells from *Nfatc2* KO mice show the hyper-proliferative response phenotype. CD4 T cells were isolated from single cell suspensions of spleens and lymph nodes from WT and *Nfatc2* KO littermates by negative immunomagnetic selection. CD4^+^CD25^−^CD44^dim^ (naïve Tconv) cells were enriched by sorting to deplete CD4^+^CD25^+^ (Treg) and CD4^+^CD44^bright^ (memory) cells. Naïve Tconv cells were labeled with CFSE, mixed 1∶1 with syngeneic AgPCs and stimulated with anti-CD3. Proliferation was measured by CFSE dilution in CD4 T cells using flow cytometry after of 96 hr of culture. (A) Representative one-dimensional histograms of CFSE dilution from unstimulated (top) or from stimulated (bottom) WT and *Nfatc2* KO cells. Insets show two-dimensional contour plots of CD4 (y-axis) and CD44 (x-axis) expression. The box indicates the percent of CD4^+^CD44^+^ cells at the end of the culture period. (B) Means ± S.D. of the percent of cells in triplicate samples that underwent 0–7 divisions over 96 hr for each genotype from 2 experiments for each genotype.

The CD44^bright^ memory compartment includes long-lived cells that undergo activation more rapidly, have a lower signaling threshold than naïve cells in response to stimulation, and are relatively resistant to Treg inhibition [25], [26]. Thus, it was possible that the altered T cell frequency in *Nfatc2* KO mice was due specifically to increased production or increased accumulation of these CD44^bright^ memory cells. To determine if *Nfatc2* deficiency also affected naïve cells, we examined proliferation in naïve (CD44^dim^) cells by CFSE dilution. Figure 5 shows naïve (CD44^dim^) CD4 Tconvs from young *Nfatc2* KO mice have a reduced threshold of activation like that seen in unseparated CD4^+^ Tconvs from *Nfatc2* KO mice, with a comparable relative enhancement in population doublings (p = 0.05 at division #3). Such short-term proliferation, however, was not sufficient to generate a greater relative frequency of CD44^bright^ memory cells in the *Nfatc2* KO genotype (Figure 5a, lower panels).

### The Hyper-proliferative T Cell Phenotype in Nfatc2 and Tob1 Knockout Mice is not Due to Treg Dysfunction

Despite the similar proportions and numbers of Treg cells in the KO mice, defective Treg function could still account for part of the expansion of activated T cells in these mice. Therefore, we examined the capacity of *Nfatc2* KO and *Tob1* KO Tregs to inhibit WT CD4^+^ Tconvs. Our results were somewhat surprising, showing that the *in vitro* suppressive activity of *Nfatc2* KO Tregs was equivalent to that of WT Tregs, but the activity of *Tob1* KO Tregs was superior (p<0.01; Figure 3B–C). Previous studies showed that pharmacological inhibition of Nfat activity alters Treg function *in vitro* [27], [28], suggesting this effect might be mediated by proteins distinct from Nfatc2. In at least one other report, however, pharmacological inhibition of NFAT did not inhibit Tregs [29]. To evaluate this in our system, we incubated WT Tregs with CsA (1 µg/ml) for 1 hr and thoroughly washed them prior to addition to the suppression assay. Figure 3B–C shows that CsA appeared to further enhance the suppressive activity of WT Tregs, suggesting that while under these conditions *Nfatc2* deficiency did not appreciably alter Treg function, the activity of other Nfat subunits might dampen Treg activity [16].

Finally, we examined if the intrinsic proliferation of naïve *Nfatc2* KO Tconvs was due to resistance to Treg-mediated suppression. WT and *Nfatc2* KO Tregs had comparable potency in suppression of naïve Tconv cell proliferation from both the WT and *Nfatc2* KO genotypes (data not shown), suggesting that the hyper-proliferative phenotype of *Nfatc2* KO T cells is probably due to defective intrinsic negative regulation and not to resistance to extrinsic inhibition.

### Nfatc2 Deficiency Leads to Enhanced Proliferation in a Lymphodepleted Environment of Homeostatic Expansion, but does not Provide Improved Survival Fitness in a Competitive Lymphoreplete Environment

A preponderance of data suggests that Nfatc2 intrinsically dampens Tconv responses and prevents persistence and accumulation of activated and memory T cells. However, the potential for *Nfatc2* KO cells to expand in a competitive WT environment has not been examined in detail. We showed previously that *Tob1*-deficient T cells expanded briskly under conditions of lymphopenia-induced proliferation (2 weeks after adoptive transfer into lymphodepleted SCID hosts). This response was more robust than that seen upon adoptive transfer of WT cells [21], suggesting that cells with defective negative regulation might be able to outcompete WT cells *in vivo*.

**Figure 6:**
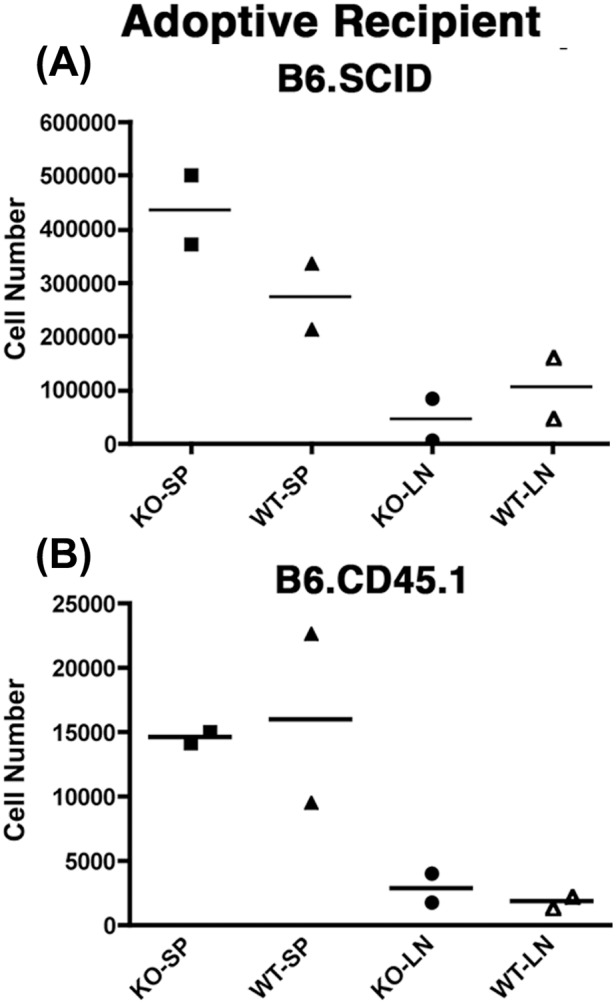
*Nfatc2* deficiency leads to enhanced proliferation in a lymphodepleted environment of homeostatic expansion, but does not provide improved survival fitness in a competitive lymphoreplete environment. CD4^+^CD25^−^ Tconvs were prepared as described from WT and from *Nfatc2* KO mice. Sorted cells (100,000) were adoptively transferred into the tail vein of two B6.SCID (top) or two B6.CD45.1 (bottom) mice. Recipients were sacrificed 15 days later and total cell numbers recovered from spleens (SP) and lymph nodes (LN) were enumerated using a CellDyn 3500 hematology analyzer. The percent of donor cells in each recipient was then calculated based on the percent of CD4^+^CD45.2^+^ cells present in each preparation, and is represented by a symbol in the graphs. Lines indicate the mean for each recipient group.

Thus, we examined if *Nfatc2* KO cells would show an advantage for growth or survival in a lymphodepleted environment. We adoptively transferred 10^5^ congenic, naïve WT Tconvs or 10^5^ *Nfatc2* KO CD4^+^CD25^−^ Tconvs into B6-SCID hosts and quantified the number of CD4^+^ T cells in recipient spleens and lymph nodes 15-days after transfer. The recipients showed no deleterious effect from adoptive transfers (*e.g.*, organomegaly or grossly evident inflammation). Figure 6A shows that greater numbers of *Nfatc2* KO donor T cells than WT T cells were recovered from the spleens, but not from the lymph nodes of adoptive recipients. One interpretation would be that *Nfatc2* KO T cells can outgrow WT T cells under conditions of homeostatic expansion. While we cannot formally rule out the possibility that *Nfatc2* KO cells simply showed preferential homing to spleen, we believe this is unlikely based the absence of splenomegaly in the *Nfatc2* KO B6 mice.

We next repeated this experiment using lymphoreplete WT hosts. Injection of 10^5^ cells into congenic (CD45.1) B6 mice showed no difference in the recovery of WT or *Nfatc2* KO donor T cells from recipient spleens and lymph nodes over a 15-day period (Figure 6B). This suggests the rate of expansion and survival was similar for both cell types. Similarly, when equal numbers of WT and *Nfatc2* KO T cells were co-injected into B6 recipients, there were no differences in recovery of these cells, suggesting *Nfatc2* deficiency did not provide an acute competitive growth or survival advantage in the lymphoreplete environment (data not shown).

### Nfatc2 KO H2^b^ Mice Develop Systemic Lymphocytic Inflammation and Plasmablastic Malignancies

**Figure 7:**
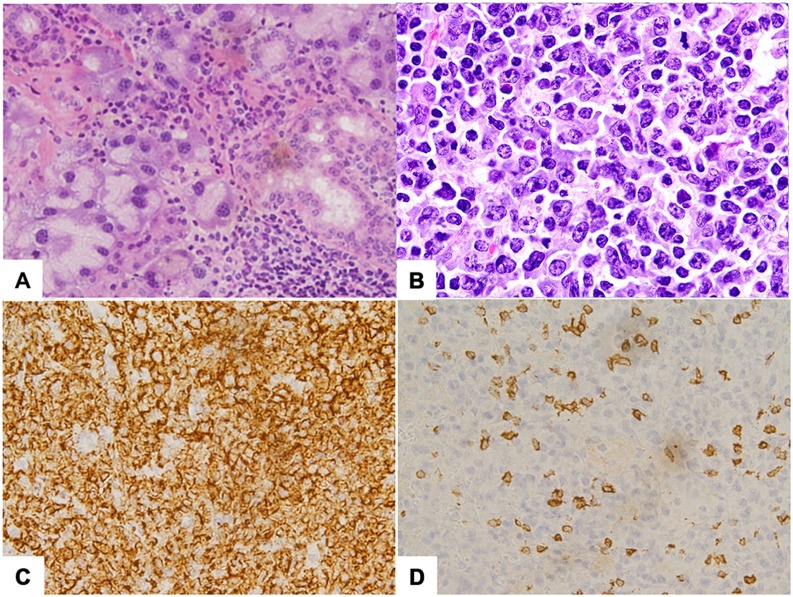
Lymphocytic infiltrates into parenchymal organs and B-cell malignancies in *Nfatc2* KO mice. (A) Photomicrograph of a representative section from the parotid salivary gland of a 21-month old *Nfatc2* KO mouse stained with H&E (magnification 100X) showing mild to moderate lymphocytic aggregates in the interstitium. Approximately 70% of the infiltrate consisted of B cells. (B) Photomicrograph of a representative section from lymph node of a 20-month old *Nfatc2* KO mice stained with H&E (magnification 400X) showing effacement with corticomedullary architecture expanded by a homogenous population of large lymphocytes with large eccentric nuclei containing open to marginated chromatin, single to multiple prominent nucleoli, and scant to moderate amphophilic cytoplasm. The section contains scattered necrotic cells and 0–2 mitoses per high power field. The histological appearance is consistent with plasmablastic plasmacytoma. Similar tumors were identified in lymph nodes, livers, and spleens of *Nfatc2* KO mice. (C and D) Immunohistochemical staining using antibodies against B220 (C) and CD3 (D) in serial sections from (B), showing the tumor is comprised by a monotonous population of malignant B cells with few residual T cells.

**Figure 8:**
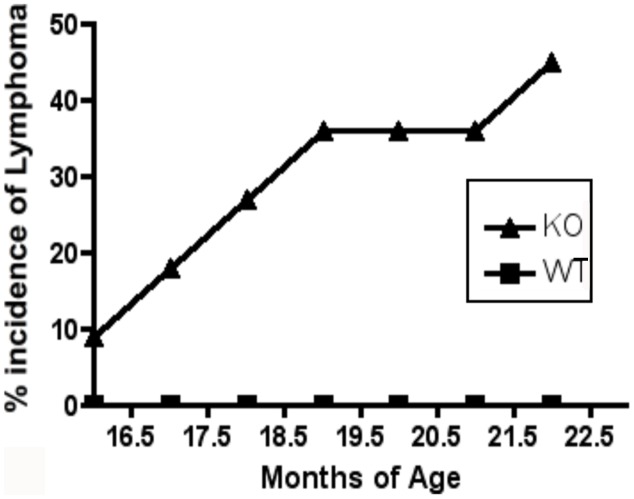
Incidence of lymphoma in *Nfatc2* KO mice. Seven of fifteen *Nfatc2* KO mice (congenic generations 6–9 from heterozygous matings) that were allowed to reach 16 to 20 months of age (denoted by triangles/KO) developed B-cell malignancies. By comparison, none of 12 wild type or *Nfatc2* age-matched hemizygous littermates (denoted by squares/WT) developed lymphoma.

Unlike the gross splenomegaly that develops in mixed background *Nfatc2* KO mice [24], [30], congenic B6 *Nfatc2* KO mice developed generalized lymphadenopathy that increased in severity with age with only mild or no splenomegaly being observed up to ∼15 months of age. However, there was frequent infiltration of T cells and B cells into the parenchymal regions of salivary glands (Figure 7A), as well as the kidney, liver, and retro-orbital tissues (data not shown). Mice older than 16 months of age also developed B cell lineage neoplasms involving the lymph nodes, spleen, liver and pancreas (Figure 8). The tumors exhibited features characteristic of anaplastic and/or plasmablastic plasmacytomas [23], [31] often with high mitotic and apoptotic indices and varying amounts of histiocytic infiltration (Figure 7B–D and data not shown). PCR-based clonality assays evaluating diversity of antigen receptor rearrangements confirmed that the tumors were derived from clonal B cell populations with residual polyclonal T cells. This age-associated tumor phenotype was not seen in any of 12 age-matched WT (*Nfatc2^+/+^*, N = 6) or hemizygous (*Nfatc2^+/−^*N = 6) littermates, and unlike previous reports [18], no tumors developed in young or old *Tob1* KO mice.

We next determined if dual deficiencies of *Nfatc2* and *Tob1* would exacerbate the parenchymal lymphoid infiltration or accelerate tumor development. Mice (N = 29 total) were humanely sacrificed at 140–150 days (5–5.5 months) of age and examined for gross changes and for microscopic abnormalities in secondary lymphoid organs, liver, and salivary glands (the latter were a common site of lymphoid infiltrates in *Nfatc2* KO mice). We did not observe any tumors in WT (N = 3), *Nfatc2* KO (N = 3), *Tob1* KO (N = 5), or *Nfatc2/Tob1* DKO (N = 6) mice by 140–150 days of age. Salivary glands from one of *Nfatc2/Tob1* DKO mice examined microscopically showed lymphoid infiltration into the parenchyma, but this was no more severe than that observed in single *Nfatc2* KO mice (Figure 7), suggesting *Tob1* does not provide a compensatory mechanism to prevent or delay the lymphoid hyper-reactivity and tumor development observed under conditions of *Nfatc2* deficiency.

## Discussion

We examined the role of Nfatc2 and Tob1 in Tconv cell proliferation and Treg function using germ line KOs in the B6 background. Our experiments support previous observations regarding the context-dependent function of Nfatc2 and Tob1 in different genetic backgrounds [14], [30], [32], [33]. *Nfatc2* KO mice in a mixed B6×129/SvJ background showed splenomegaly with reduced thresholds for T-cell activation, and these observations were faithfully replicated in our studies of the same mice [24]. We then introduced the *Nfatc2* KO mutation into the B6 background using the speed congenic approach based on informative microsatellites and on the absence of MLR with the parental B6 strain. The most striking difference between the *Nfatc2* KOs in the pure B6 vs. the mixed genetic background was the absence of splenomegaly in the former; this remains a consistent finding after breeding these KO mice for more than 20 generations. Despite these phenotypic differences, splenic and lymph node CD4^+^ Tconv cells showed similar hyper-responsiveness on both genetic backgrounds. The development of tumors in excess of that seen in the WT controls for the *Nfatc2* KO strain also had not been a previously reported finding. However, this may be because in other studies, the mice had not been allowed to age to the time these tumors developed (G. Crabtree, A. Rao, personal communication). Consistent with previous observations [20], *Tob1* KO mice did not exhibit gross immunologic phenotypes through 20 months of age, but the *Tob1* KO mice in our colony did not show an increased propensity for spontaneous tumor development.

The mechanisms underlying the propensity for preferential development of plasma cell neoplasms remain to be examined. *Nfatc2* acts as a negative regulator of proliferation in hematopoietic and other cell types, including B cells [5], [34], and we observed persistently activated memory B cells in *Nfatc2* KO mice. In this regard, it is of interest that gene expression analysis of anaplastic and plasmablastic plasmacytomas from other mouse strains indicated that they originated from memory B cells [31]. Recent experiments showed that *Nfatc2* deficiency in the background stroma impaired establishment of B16 melanoma tumors in the lung, suggesting a role for this protein in tumor-associated inflammation [35]. In addition, *Nfatc2* deficient T cells also were resistant to tumor-induced clonal anergy in the B16 model [11]. Together, these data indicate that the absence of Nfatc2 creates an environment where the potential loss of intrinsic tumor suppressive function is countered by an unfavorable environment for tumor formation. This in turn provides a potential explanation for the long latency that precedes tumor formation in *Nfatc2* KO mice.

Nfatc2 and Tob1 probably do not have redundant negative regulatory functions in T cells. Mice from both KO strains develop hyper-responsive T cells. However *in vivo*, *Nfatc2* deficiency leads to a progressive and accelerated accumulation of persistently activated memory cells, which is less evident in the case of *Tob1* deficiency. Accumulation of memory T cells in *Nfatc2* KO mice was reported previously [32], although the mechanism remains poorly understood. Induction or maintenance of T cell anergy by Nfatc2-regulated signaling molecules explains the reduced threshold for signaling observed in *Nfatc2*-deficient cells [36], but it cannot completely explain the accumulation of persistently activated memory cells. Conversely, the roles of Nfatc2 to maintain quiescence and promote cell cycle exit [24] may provide an explanation for the discrepant phenotypes seen in *Nfatc2* KO and *Tob1* KO mice.

The hyper-proliferative phenotype in *Nfatc2* KO mice was only partly due to the accumulation of memory T cells, as stringently-sorted naïve (CD44^dim^) T cells from *Nfatc2*-deficient mice proliferated more readily than cells from WT mice. Curiously, a previous study showed that the intrinsic negative regulatory function of Nfatc2 in mice with mixed MHC background was confined to CD8^+^ T cells and was not required for tolerance induction or regulatory function of CD4^+^ T cells [37]. In contrast, our data indicate that in the H2^b^ background, Nfatc2 unequivocally plays a role in restraining proliferation of CD4^+^ T cells. It remains to be determined if these differences can be used to dissect discrete functions of Nfatc2 to maintain quiescence (cell cycle exit) and tolerance, or if they are due to differences mediated by MHC-dependent signals.

It has been reported that Nfat proteins are required to generate functional Tregs, as they interact with Foxp3 and other Treg-specific or Treg-selective factors that regulate expression of IL-2, CD25, CTLA-4, and Foxp3 itself [5], [7], [10], [36]. However, recent data have challenged the requirement for Nfatc2 to support Treg function [15], and suggested it is the combined threshold of Nfat proteins (all isoforms), which regulates Foxp3 expression and generation of iTregs [16]. Furthermore, ablation of *Nfatc2* and *Nfatc3* is associated with resistance of CD4 Tconvs to Treg-mediated suppression [10]. In our experiments, neither deficiency of *Nfatc2* nor *Tob1* by itself altered the frequency or the phenotype (CD4/CD25/Foxp3/CTLA-4/CD126) of Tregs in peripheral lymphoid organs compared to WT littermates. *Nfatc2*-deficient Tregs had comparable suppressive function to WT Tregs, and naïve *Nfatc2* KO Tconvs were not resistant to the suppressive effects of Tregs. So, even though *in vitro* Treg activity may not always reflect *in vivo* Treg activity, our data suggest that accumulation of persistently activated memory T cells in the B6 *Nfatc2* Kos might not be simply due to Treg failure or resistance to Treg suppression. Somewhat surprisingly, *Tob1*-deficient Tregs were more potent that either WT or *Nfatc2* KO Tregs. This could explain why *Tob1* KO mice have fewer persistently activated and memory cells *in vivo*, as their Tregs might help restrain activation of intrinsically hyper-reactive Tconvs.

In our experiments, pharmacological inhibition of Nfat proteins in Tregs by CsA appeared to enhance *in vitro* suppressive function. This result differs from that of previous studies showing that CsA inhibits human Treg function by reducing secretion of IL-2 and IFN-gamma without affecting expression of IL-10 or TGFβ [38]. This observation was made repeatedly for Treg cells [27], [39], [40], but was not always the case [29]. Furthermore, the effect appears to be dose dependent, and paradoxically apparent at lower (albeit therapeutic) doses of CsA [28], [38]. We did not test inhibition of WT Tregs at lower doses of CsA (*e.g.*, 40 ng/ml), but the *Nfatc2* KO mice in the B6 background provide one model to study CsA-dependent effects on Tregs in future experiments.

The hyper-proliferative phenotype of naïve *Nfatc2* KO CD4^+^ Tconvs was maintained under conditions of homeostatic expansion, as we showed previously for *Tob1* KO T cells [21]. However, the effect was relatively small (at least over a two week time for expansion), and the *Nfatc2*-deficient cells showed no competitive advantage when they were adoptively transferred to lymphoreplete wild type B6 mice. This suggests that any advantages of accelerated growth may be reduced under conditions of rapid proliferation such as those found in cells undergoing homeostatic expansion. It also implies that the accumulation of persistently activated T cells in *Nfatc2* KO mice may depend on complete absence of this protein in the host genetic background, or, alternatively, that it may be partly adaptive or regulated by other variables in the microenvironment including Tregs [5] and/or the stromal cells that support proliferative niches [35]. On the other hand, longer assays may be required to reveal a competitive advantage of *Nfatc2* KO T cells in lymphoreplete WT hosts, as well as to assess if these cells have heightened inflammatory or autoimmune potential in Treg-deficient lymphodepleted hosts.

With regard to tumor development, we favor the explanation that *Nfatc2* deficiency facilitates development of plasmablastic malignancies similar to those seen in *Fasl*-deficient (*gld*) mice and other mice [31], [41], which might be driven by chronic antigenic stimulation or perhaps by autoantigens [42]. This could be a consequence of poorly restrained intrinsic negative regulation and persistent cell cycle transit by lymphocytes, despite the potential absence of a promiscuous inflammatory milieu. Antigen receptor-driven apoptosis of transformed B cells (Burkitt lymphoma) also has been shown to be a consequence of Nfatc2-mediated signals [43], suggesting loss of Nfatc2 could provide a survival advantage to malignant B cells. Thus, *Nfatc2* KO mice could represent a valuable resource to explore mechanisms that account for progression from chronic antigenic stimulation to malignancy [44].

In conclusion, our data are consistent with recently published experiments by Macian’s group in a tumor-induced anergy system [11] in showing that intrinsic negative regulation of T-cell proliferation is a prominent role for Nfatc2, and its duality as a master controller of Tregs is less evident in the B6 background. These activities seem to be distinct from and non-redundant with the role of Tob1, whose intrinsic negative regulatory function extends to Tregs, and which appears to have a prominent function in restraining autoreactivity [21], [22]. Further studies are needed to assess the potential for selective modulation of Nfatc2, Tob1, or both as part of therapeutic strategies seeking to alter T cell responses in patients with autoimmune diseases and cancer.
